# Effects of Good Pain Management (GPM) ward program on patterns of care and pain control in patients with cancer pain in Taiwan

**DOI:** 10.1007/s00520-020-05656-x

**Published:** 2020-08-15

**Authors:** Wei-Chih Su, Chieh-Han Chuang, Fang-Ming Chen, Hsiang-Lin Tsai, Ching-Wen Huang, Tsung-Kun Chang, Ming-Feng Hou, Jaw-Yuan Wang

**Affiliations:** 1 Division of Colorectal Surgery, Department of Surgery, Kaohsiung Medical University Hospital, Kaohsiung Medical University, No. 100 Tzyou 1st Road, Kaohsiung, 807 Taiwan; 2 Department of Surgery and ICU, Kaohsiung Municipal Siaogang Hospital, Kaohsiung, Taiwan; 3 Division of Breast Surgery, Department of Surgery, Kaohsiung Medical University Hospital, Kaohsiung Medical University, Kaohsiung, Taiwan; 4 Department of Surgery, Kaohsiung Municipal Ta-Tung Hospital, Kaohsiung, Taiwan; 5 Department of Surgery, Faculty of Medicine, College of Medicine, Kaohsiung Medical University, Kaohsiung, Taiwan; 6 Graduate Institute of Clinical Medicine, College of Medicine, Kaohsiung Medical University, Kaohsiung, Taiwan; 7 Graduate Institute of Medicine, College of Medicine, Kaohsiung Medical University, Kaohsiung, Taiwan; 8 Center for Cancer Research, Kaohsiung Medical University, Kaohsiung, Taiwan; 9 Cohort Research Center, Kaohsiung Medical University, Kaohsiung, Taiwan; 10 Master Program for Clinical Pharmacogenomics and Pharmacoproteomics, School of Pharmacy, Taipei Medical University, Taipei, Taiwan

**Keywords:** Good pain management, Pain control, Adequacy of pain treatment, Cancer pain

## Abstract

**Background:**

The undertreatment of cancer pain is a global issue although many international guidelines and various studies bloom to explore the approaches in pain management. However, there is no standard care for cancer pain in routine practices. To set up a standardized procedure for improving cancer pain management in Taiwan, the Good Pain Management (GPM) program is explored to provide treatments following the US National Cancer Care Network (NCCN) Adult Cancer Pain Guideline.

**Method:**

Patients diagnosed with moderate-to-severe cancer pain were eligible and randomized into the GPM or control arm and observed the first 48 h to evaluate the effects of pain management between 2 arms. Pain control, adequacy of treatments, patient satisfaction, and quality of life (QoL) of eligible patients were analyzed. Ad hoc analyses based on the pain medication category were also conducted.

**Result:**

Fifty-one patients were enrolled, with 26 and 25 assigned to the GPM and control arms, respectively. Significant differences among the GPM and control arms were found including a greater decrease in the mean numerical rating scale (NRS) score in the GPM arm (− 4.6 vs. − 2.8), a lower proportion of moderate-to-severe pain in the GPM arm (23.2% vs. 39.8%), and a higher pain management index (PMI) score in the GPM arm (0.64 points vs. 0.33 points) (all *p* < 0.05). Ad hoc analyses revealed that the patient subgroups using strong opioids showed better patient satisfaction in GPM arm when compared with the same subgroup in the control arm.

**Conclusion:**

In summary, our study demonstrated that the implementation of a standardized pain assessment and management approach (GPM ward program) showed significant improvements on pain relief, decreased the portion of moderate-to-severe cancer pain, and increased patient satisfaction in the 1st 48 h after admission. The implementation of the GPM approach in the cancer ward may provide sooner and better improvement of cancer pain management for patients who suffered moderate-to-severe cancer pain.

**Trial registration:**

ClinicalTrials.gov (Identifier: NCT03155516)

## Introduction

Pain is one of the most common symptoms occurring in 53% of cancer patients in all stages of the disease, and over 70% of patients in advanced stages experience uncontrolled pain of moderate-to-severe intensity worldwide [1–3]. Also, unrelieved pain is highly associated with affecting emotion, activity, and the quality of life for patients [4–6]. Although there are international guidelines for pain management [7–10], the undertreatment of cancer pain is still a widespread issue in Asia, Europe, and North America for 59.1%, 40.3%, and 39.1% of cancer patients, respectively [2]. Okuyama et al. [11] and Vuong et al. [12] focused on the evaluation of adequacy for pain management using the pain management index (PMI) [13] and results showed that pain management needed to be improved based on a high proportion of patients with negative PMI (~ 70% of Japanese patients and 33% of Canadian patients). Hence, implementing effective pain management for the current clinical practice has become a crucial target around the world.

Results from several investigations advocate the implementation of a standardized pain management plan. In America, various randomized studies were conducted for the assessment of effects on the education for pain in cancer patients and results showed a decrease in pain intensity following the education [14–16]. Additionally, the Good Pain Management (GPM) ward program implemented in China improved the quality of life (QoL) for patients [17, 18].

**Fig. 1:**
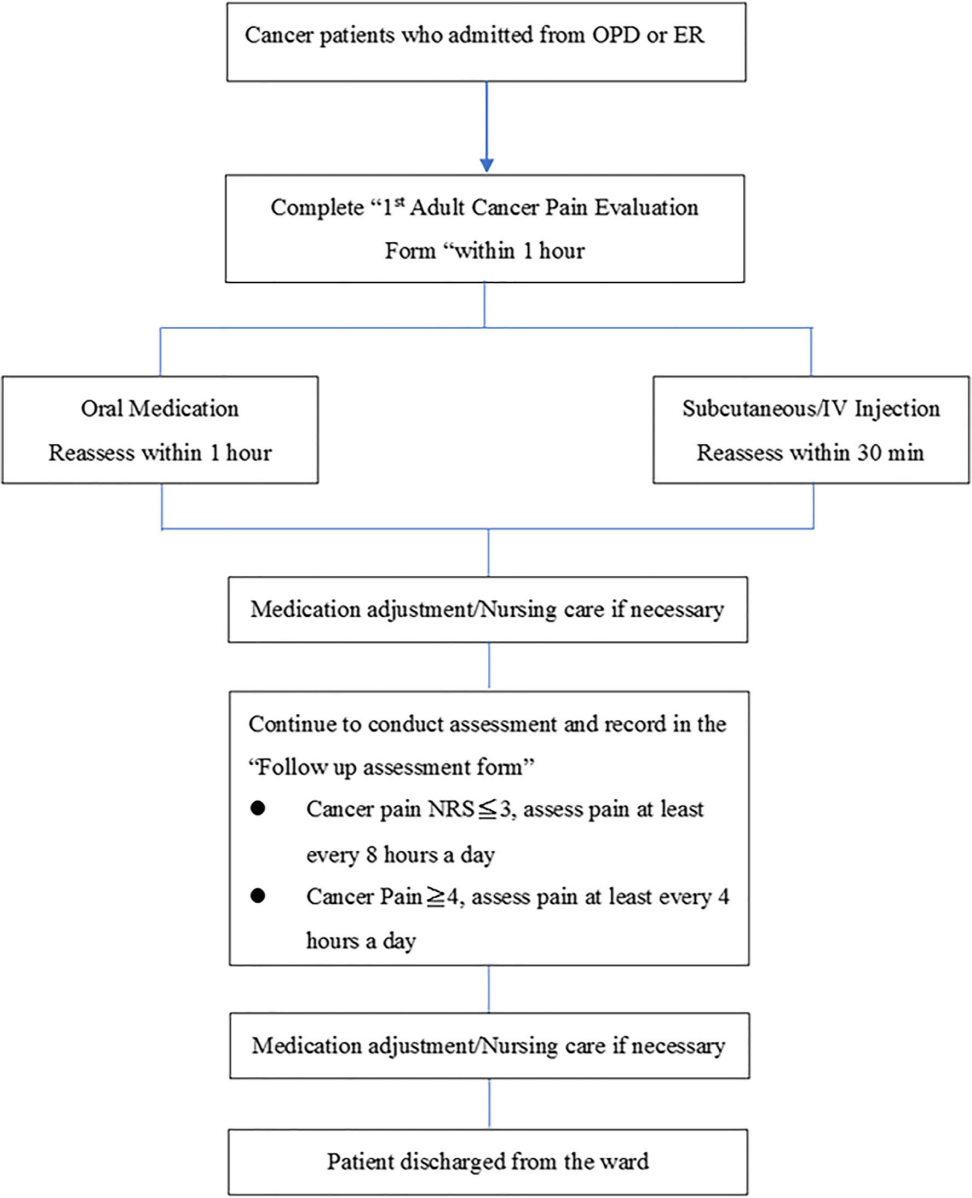
Good pain management—algorithm

Nevertheless, a Taiwanese population–based study revealed that 23.6% of cancer patients had inadequate pain medication and 47.4% were not satisfied with the pain relief during admission [19]. Comparable results were observed in another study [20] despite the relevant regulation of pain control launched in 2005 [3]. To standardize pain management in Taiwan, the study aimed to set up the GPM ward with streamlined assessment and management procedures. The program enforced regular pain assessments during the admission, with adequate treatments administered to patients with moderate-to-severe cancer pain (i.e., strong-opioids) based on the National Cancer Care Network (NCCN) Adult Cancer Pain Guideline [8]. Patients in this study were recruited based on the good pain management algorithm (Fig. 1)

This study aimed to investigate the benefits and effects of optimal pain control within the first 48 h after cancer patients with moderate-to-severe cancer pain were hospitalized and planned to demonstrate the viability of the GPM ward in daily practices in Taiwan.

## Material and methods

### Study design and procedure

This randomized, current practice-controlled study was approved by the Institutional Review Board of our Hospital (KMUHIRB-F(II)-20160086) and registered on ClinicalTrials.gov (Identifier: NCT03155516) before enrolling study participants. Adult patients with moderate-to-severe cancer pain and hospitalized over 24 h were eligible and randomized into either GPM or control arm in a 1:1 ratio. Patients were excluded if they had non-cancer pain or unexplained pain based on the evaluation of their medical history, moderate-to-severe mental disorder, or received surgery within 24 h before admission. Data was collected in approximately 48 h after the entry of this study to evaluate the pain management status of the enrolled patients with moderate-to-severe cancer pain. In the control arm, the pain management for patients was performed under the routine practice with less regular assessments (once every 8 h) and reassessments after rescue medication provision. In the GPM arm, patients were evaluated by brief pain inventory (BPI) within 1 h after admission and treated with analgesics based on a numerical rating scale (NRS) [21, 22]. The pain level was closely monitored using NRS and medication will be adjusted accordingly if the patients’ NRS > 4 and above. Patients with pain intensity of ≤ 3 points were monitored once at least every 8 h and those with pain intensity of ≥ 4 points were monitored at least once every 4 h until discharge.

### Study assessments

The pain control, adequacy of pain treatments, patients’ satisfaction, and QoL were compared between the GPM and control arms. The pain intensity was evaluated using NRS and classified into none (0 points), mild (1–3), moderate (4–6), and severe (7–10). The association between pain intensity and adequacy of analgesics was estimated by PMI [13]. Moreover, the American Pain Society Patient Outcome Questionnaire (APS-POQ) was used to survey the first 24 h of satisfaction and outcomes after 48 ± 8 h of admission.

Ad hoc subgroup analyses based on the pain medication category were also conducted. Two subgroups (strong opioids and non-strong opioids) in both GPM and control arms were divided and parameters such as pain control, patient satisfaction, and QoL were analyzed and compared.

### Treatment procedure

After the randomization, patients in the control arm received treatments following routine practice for pain management, while patients in the GPM arm received analgesics according to pain levels classified by NRS. Non-opioids (e.g., acetaminophen/non-steroidal anti-inflammatory drug, NSAIDs) were given to manage mild pain, and weak/low-dose strong opioids or strong opioids (e.g., morphine, oxycodone, and fentanyl) were given to manage moderate or severe pain. All analgesics were introduced at a tolerable frequency and dosage per physicians’ judgments.

### Statistical analyses

Continuous variables were described using the number of observations, mean, median, standard deviation (SD), and 95% confidence intervals (95% CI). Changes from baseline were analyzed by paired *t* test or Wilcoxon signed rank test based on the normality assumption, and the difference between groups was compared by two-sample *t* test for normal distribution or the Wilcoxon rank sum test for non-normal distribution. Categorical variables were tabulated as frequency and percentage and compared by the chi-square test or Fisher’s exact test as appropriate between groups. The pain reporting outcome was presented as a proportion of different pain intensity among all events. The significant difference was defined by two-sided *p* value < 0.05. Data analysis was performed using SAS® statistical software (SAS Institute, Cary, NC, USA) 9.4.

## Results

### Patient demographic

**Fig. 2:**
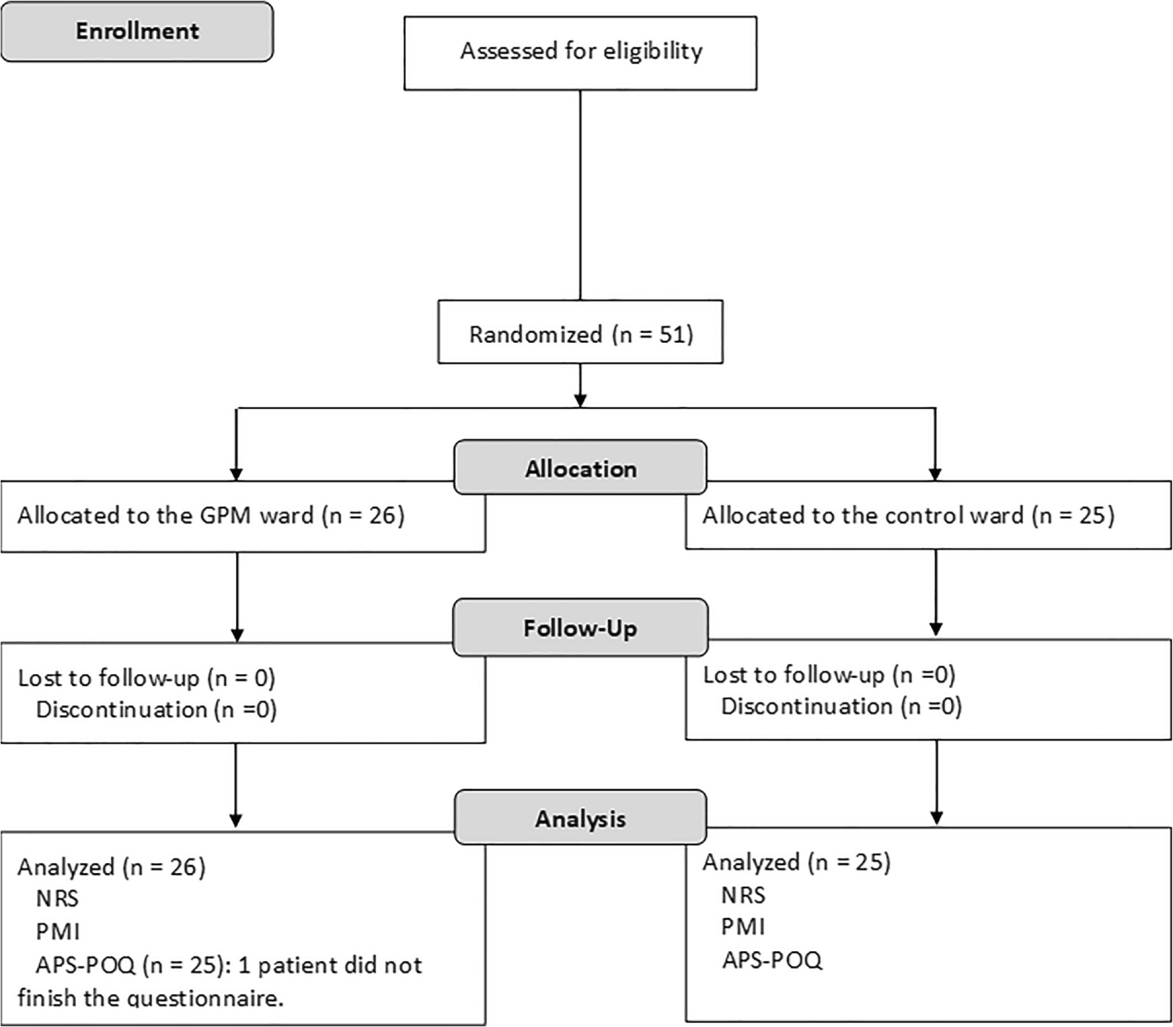
Flow chart

A total of 51 patients were enrolled from the end of 2016 to August 2017 and randomized to the GPM arm (*n* = 26) or the control arm (*n* = 25) (Fig. 2). Table 1 summarizes the demographics and pain characteristics. Overall, no significant difference was observed between groups and most patients experienced moderate cancer pain. Patients were around 52–56 years old and approximately 36% of them were male. More than 70% of the patients suffered from cancer metastasis with multiple sites.

**Table 1:** Demographics and pain characteristics of enrolled patients

Variables	GPM arm, *n* = 26	Control arm, *n* = 25	*p* value^a^
Age (years, mean ± SD)	52.2 ± 14.3	56.2 ± 13.6	0.31
Male, *n* (%)	9 (34.6)	9 (36.0)	> 0.999
NRS score (mean ± SD)	6.4 ± 1.7	5.4 ± 1.3	0.03*
Cancer type	26	25	
Colorectal	18	13	
Gastric	3	3	
Breast	2	3	
Others	3	6	
Metastasis			
No (%)	3 (11.5%)	9 (36%)	
Liver	8	5	
Lung	5	4	
Peritoneal	4	3	
Ovary	3	2	
Abdominal wall	3	2	
Lymph node	2	4	
Bowel	1	1	
Bone	2	2	
Presacral	1	0	
Skin	1	1	
Pain level, *n* (%)			
Moderate	19 (73.1)	20 (80.0)	0.56
Severe	7 (26.9)	5 (20.0)	

^a^The difference between groups was analyzed by two-sample *t* tests for the continuous data and chi-square test or Fisher’s exact test for the categorical data

*Significant difference

*Abbreviations*: *GPM*, good pain management; *NRS*, numerical rating scale; *SD*, standard deviation

### Pain control

**Fig. 3:**
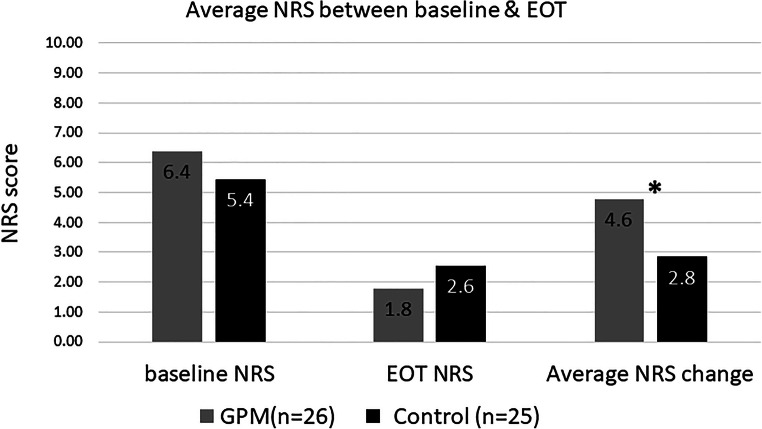
A comparison of change in the NRS score between the GPM and control arms. NRS, numerical rating scale; GPM, good pain management; EOT, end of treatment. *p = 0.0013

**Fig. 4:**
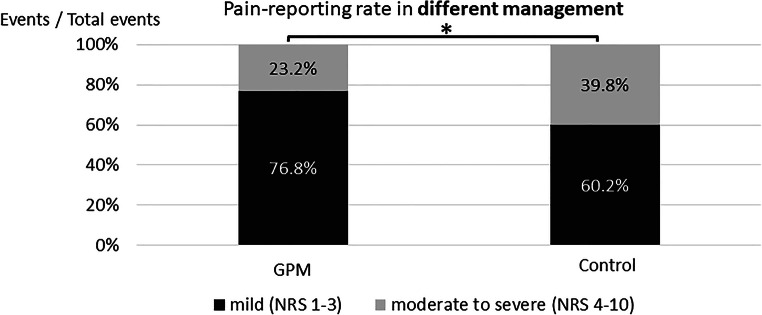
The frequency of pain reported in the GPM and control arms. GPM, good pain management. *p < 0.0001

The mean change from baseline in the NRS score is presented in Fig. 3. After 48-h pain management, the mean change in NRS score was significantly higher for the GPM ward (− 4.6) compared with that for the control ward (− 2.8) (*p* = 0.0013). The frequency of pain reporting stratified by pain levels is shown in Fig. 4. The percentage of moderate-to-severe pain events in the GPM ward was significantly lower than in the control ward (23.2% vs. 39.8%; *p* < 0.0001).

### Adequacy of pain treatment

The summary of analgesic administration following different pain management is tabulated in Table 2. For patients with moderate-to-severe pain, the prescription rate for strong opioids was higher in the GPM ward than in the control ward (70.7% [41/58] vs. 63.3% [31/49]). The mean PMI score was significantly higher for the GPM ward than for the control ward (0.64 vs. 0.33, *p* = 0.0343), suggesting that patients in the GPM ward were more likely to receive adequate pain relief.

**Table 2:** Treatment prescribed for 51 cancer patients

Treatment for pain control	Pain levels during admission, events (%)									
	GPM group					Control group				
	No pain	Mild	Moderate	Severe	Total	No pain	Mild	Moderate	Severe	Total
No treatment	0 (0.0)	0 (0.0)	0 (0.0)	0 (0.0)	0 (0.0)	0 (0.0)	0 (0.0)	2 (5.4)	0 (0.0)	2 (3.9)
Non-opioid	0 (0.0)	1 (14.3)	0 (0.0)	0 (0.0)	1 (1.5)	0 (0.0)	0 (0.0)	2 (5.4)	0 (0.0)	2 (3.9)
Weak opioid	0 (0.0)	1 (14.3)	16 (35.6)	1 (7.7)	18 (27.3)	0 (0.0)	0 (0.0)	13 (35.1)	1 (8.3)	14 (27.5)
Strong opioid	1 (100.0)	5 (71.4)	29 (64.4)	12 (92.3)	47 (71.2)	0 (0.0)	2 (100)	20 (54.1)	11 (91.7)	33 (64.7)
Total	1 (1.5)	7 (10.6)	45 (68.2)	13 (19.7)	66	0 (0.0)	2 (3.9)	37 (72.5)	12 (23.5)	51

*Abbreviation*: *GPM*, good pain management

### Effects on patients’ quality of life

**Fig. 5:**
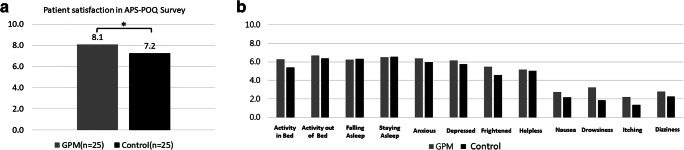
The difference between the GPM and control arms using APS-POQ. **a**. Patient satisfaction *p = 0.724. **b** Overall outcomes. APS-POQ, American Pain Society Patient Outcome Questionnaire; GPM, good pain management. All *p* > 0.05

Figure 5 illustrates the APS-POQ outcomes regarding patients’ satisfaction and QoL. In general, patients’ satisfaction and QoL in two arms were comparable (both *p* > 0.05) and had no significant difference.

### Ad hoc analysis based on the pain medication category

**Fig. 6:**
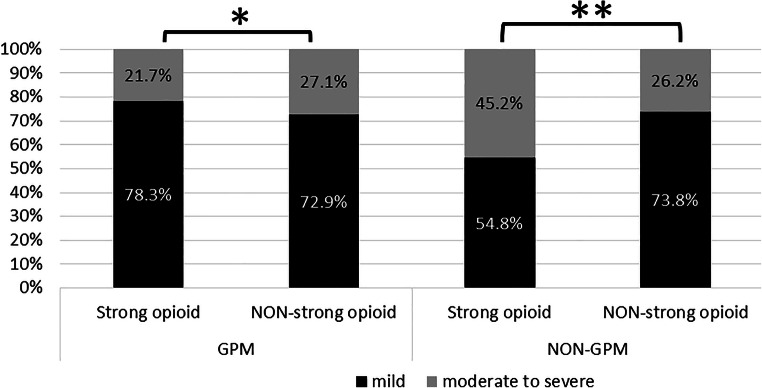
The frequency of pain reporting in the GPM and control arms—ad hoc subgroup analysis based on the pain medication category (strong opioid group and non-opioid group). *p = 0.4046; **p = 0.0132, chi-square test

To have a deeper understanding of the results from the study, an ad hoc analysis was conducted. Based on the pain medication that the patients were prescribed, the data of the 2 patient groups (strong opioid group and non-strong opioid group) were divided for further analysis. Thirty-seven patients were identified as a strong opioid group and 14 patients as non-strong opioid group. The ad hoc analysis of the pain control rate showed no statistical difference between the strong opioid subgroup and non-opioid subgroup in the GPM arm, while there was a significant difference in the higher moderate-to-severe pain reporting rate in the strong opioid subgroup compared with the non-opioid subgroup in the control arm (Fig. 6). This may imply the intervention of GPM will help the patients who need strong opioids to have less moderate-to-severe cancer pain. Similar results were also observed; when comparing the GPM arm with control arm in the strong opioid subgroup, a significant reduction of moderate-to-severe reporting rate in the GPM arm was noted (21.7% vs. 45.2%, *p* < 0.0001, data not shown). There was no significant difference in the moderate-to-severe reporting rate between the GPM and control arms within the non-opioid patient subgroup (27.1% vs. 26.2%, *p* = 1.0, data not shown).

**Fig. 7:**
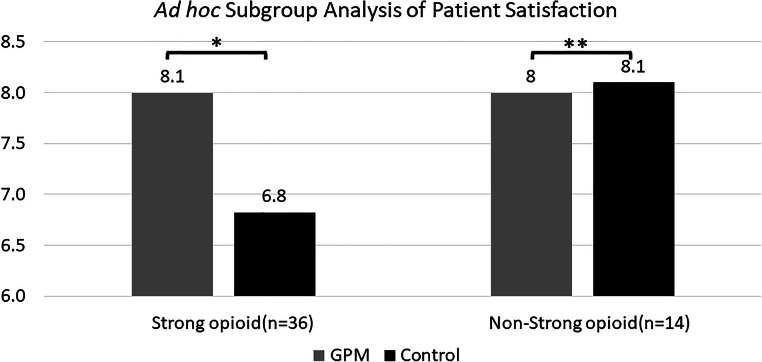
The ad hoc subgroup analysis of patient satisfaction (APS-POQ). Comparison on patient satisfaction on GPM and control ward among the same patient groups (strong opioid group and non-opioid group, *p = 0.0329; **p = 0.8697, independent two-sample t-test)

Since there was no significant difference in patient satisfaction between the GPM and control arms, another ad hoc subgroup analysis was also conducted. Significant patient satisfaction was observed in the GPM arm compared with that in the control arm when the patients were in the strong opioid subgroup (8.1 vs. 6.8, *p* = 0.0329, Fig. 7). However, no similar effect was observed between the GPM and control arm in the non-opioid patient subgroup (8 vs. 8.1, *p* = 0.8697, Fig. 7).

## Discussion

We collected the data regarding cancer pain management from 51 Taiwanese patients with moderate-to-severe pain in this randomized study. The results indicated that the implementation of the GPM program in wards provided better and sooner pain relief than routine practices within the first 48 h of patients who were admitted to the hospital with moderate-to-severe cancer pain. Compared with the control arm, the GPM arm showed a significantly greater pain reduction levels in the NRS score and reported fewer numbers of moderate-to-severe pain. Also, a higher PMI score was found in the GPM arm, although the higher number of PMI may not necessarily reflect more adequate pain management. These data suggest that cancer pain can be managed efficiently within 48 h after being admitted through the GPM approach.

This GPM program has been implemented in China and our results are similar to studies conducted in China in supporting the standardized GPM program to improve pain control [17, 18]. Yang et al. [17] revealed that the mean PMI score for the GPM ward was significantly higher than for the control arm.

However, surprisingly, our PMI results were prominently superior to those in the Chinese study regardless of GPM or control arms (Taiwan vs. China: 0.33–0.64 vs. − 0.261 to 0.0083), somehow indicating more adequate cancer pain care management in our hospital system using GPM approaches.

The assessment of the patients is always the very first step for cancer pain management [23]. Compared with other countries regarding standardized pain management, a ~ 52% reduction of NRS score in the control arm of our study was comparable with that of other studies. Under the routine care for pain in cancer patients, about 52% NRS reduction for all pain levels was observed in Thailand [24], and a 59% decrease in the NRS score was recorded in Germany [25]. Nevertheless, in the GPM arm of our study, it contributed to a higher decrease in the NRS score by 70%.

However, studies in other countries following the WHO guidelines [23, 26] to manage the cancer pain showed smaller reductions in pain levels in Italy (48%) [27], Greece (57%) [28], Brazil (50–65%) [29], and Thailand (65%) [24]. These findings demonstrated the effectiveness of the GPM approach in our hospital system. GPM approach may provide another better, standardized method for cancer pain management in the hospital cancer ward.

With respect to the influence on patients’ quality of life, no significant difference in satisfaction/QoL between the two arms was noted. The ad hoc subgroup analysis of patient satisfaction showed significant patient satisfaction observed in the strong opioid patient subgroup of the GPM arm compared with the subgroup in the control arm but no difference in the non-opioid patient group. This may be because the GPM approach can provide more pain assessments on patients who need strong opioids so that healthcare professionals can identify more patients’ needs and provide more help to relieve their cancer pain. The ad hoc analysis results were similar to the results from the GPM program in China and showed an improved satisfaction/QoL in the GPM group [30, 31].

In addition, another study conducted by Wang et al. [32] showed comparable satisfaction/QoL at the time of discharge among the GPM and control arms; however, with continuous GPM approach after discharge, satisfaction/QoL outcome was significantly improved in the GPM arm 1 month after discharge, implying that the GPM approach was not only able to improve the cancer pain management in hospital ward but also was very useful when GPM approach is performed in outpatient department and maintain patient’s outcome for a longer period of time after discharge.

Our study had some shortcomings. First, the patient number enrolled was relatively small. Second, we only followed up patients for 48 h for the study purposes and we were not able to see a long-term effect even after discharge from the hospital. Third, previous researches [2, 33] pointed out that the validity of PMI applied for measuring the quality of cancer pain management was limited because it merely considered the pain intensity and types of prescribed opioids as the determinants, excluding potential factors such as disease stage, pain characteristics, complementary therapy, and geographical area.

Based on this preliminary study, we may consider different parameters that can demonstrate more details in future studies in Taiwan. Large-scale studies focusing on 48 h and a longer follow-up period may be necessarily performed to confirm our findings in more detail. Quality-/quantity-related indicators may be identified through those studies so that they will help the HCPs to manage cancer pain in a cost-effective manner.

## Conclusion

This pilot study revealed that the implementation of the GPM ward program is viable in daily practices and effective for the improvement of cancer pain management in Taiwan, especially in the first 48 h after admission, especially for those patients with moderate-to-severe cancer pain who need strong opioids. The GPM ward program may help to optimize cancer pain management in cancer wards with clear key monitoring indicators in Taiwan.
